# Surgical outcomes after collagenase *Clostridium histolyticum* failure in patients with Peyronie’s disease in a multicenter clinical study

**DOI:** 10.1038/s41598-020-80551-3

**Published:** 2021-01-08

**Authors:** Andrea Cocci, David Ralph, Rados Djinovic, Georgios Hatzichristodoulou, Girolamo Morelli, Andrea Salonia, Paolo Capogrosso, Andrea Romano, Gianmartin Cito, Fabrizio Di Maida, Esaú Fernández-Pascual, Javier Romero-Otero, Paulo Egydio, Marco Falcone, Mirko Preto, Giovanni Chiriacò, Jack Beck, Maarten Albersen, Suks Minhas, Giovanni Cacciamani, Juan Ignacio Martinez Salamanca, Nicola Mondani, Andrea Minervini, Giorgio Ivan Russo

**Affiliations:** 1 Department of Urology, Careggi Hospital, University of Florence, Florence, Italy; 2 St. Peter’s Andrology Centre and UCLH, London, UK; 3 Sava Perovic Foundation, Center for Genito-Urinary Reconstructive Surgery, BelMedic General Hospital, Belgrade, Serbia; 4 Department of Urology, Martha-Maria Hospital Nuremberg, Nuremberg, Germany; 5 Department of Urology, Cisanello Hospital, University of Pisa, Pisa, Italy; 6 Division of Experimental Oncology/Unit of Urology, URI, IRCCS Ospedale San Raffaele, Milan, Italy; 7 Lyx Institute of Urology, Universidad Francisco de Victoria, Madrid, Spain; 8 Urology Department, Hospital Universitario, 12 de Octubre, Madrid, Spain; 9 Penile Curvature Center, São Paulo, Brazil; 10 Urology Department, Città della Salute e della Scienza-Molinette Hospital, University of Turin, Turin, Italy; 11 Department of Urology and Men’s Health, St. Antonius Hospital Nieuwegein, Utrecht, The Netherlands; 12 University Hospitals Leuven, Leuven, Belgium; 13 Department of Urology, Charing Cross Hospital, Imperial College Healthcare NHS Trust, London, UK; 14 USC Institute of Urology and Catherine and Joseph Aresty Department of Urology, University of Southern California, Los Angeles, CA USA; 15 Department of Urology, Hospital Universitario Puerta De Hierro-Majadahonda, Madrid, Spain; 16 Andrology Center, Villa Donatello Private Hospital, Florence, Italy; 17 Department of Surgery, Urology Section, University of Catania, Catania, Italy

**Keywords:** Urogenital diseases, Sexual dysfunction

## Abstract

In the present study we aimed to investigate the surgical outcomes of patients with persistent penile curvature (PC) after Collagenase Clostridium histolyticum (CCH) intraplaque injections. Data from 90 patients with persistent PC after CCH in a multicentre study from 6 andrological centres were retrospectively reviewed. Three standardized surgical techniques were performed. Group 1: plaque incision grafting (PIG) with penile prosthesis implant (PPI); Group 2: PIG without PPI; Group 3: Nesbit technique. Hospital stay, operative time, postoperative complications and PC persistency/recurrence (> 20°) were evaluated. Overall satisfaction and functional outcomes were assessed through International Index of Erectile Function-Erectile Function (IIEF-EF), Peyronie’s Disease Questionnaire (PDQ), Female Sexual Function Index (FSFI) administered pre and 3 months postoperatively. Of all, 25 (27.8%) patients received grafting procedure + PPI (Group 1), 18 (20.0%) patients belonged to Group 2, and 47 (52.2%) to Group 3. Bovine pericardium graft and collagen fleece have been used in in 22 (51.2%) and 21 (48.8%) patients, respectively. Median penile length after surgery was 13.0 cm (IQR 12.0–15.0). After surgery, Group 1 showed higher increase in penile length after surgery and better improvements in terms of PDQ-PS. In contrast, both IIEF-EF and FSFI scores did not differ among groups. Overall, 86 (95.6%) did not report any complication. 4 (4.4%) patients had PC recurrence; of those, 2 (8.0%), 1 (5.6%) and 1 (2.1%) cases were observed in Group 1, Group 2 and Group 3, respectively. In case of persistent PC after CCH, surgical correction by grafting with or without concomitant PPI or Nesbit technique emerged as a technically feasible, effective and safe procedure, with no significant postoperative complications.

## Introduction

The effectiveness of intraplaque collagenase *Clostridium histolyticum* (CCH) in reducing penile curvature (PC) and improving sexual function in men with Peyronie’s disease has been demonstrated in numerous clinical studies^1–3^, In this context, Cocci et al.^4^ reported a significant efficacy in up to 57% of their cohort of patients, with baseline PC (odds ratio [OR] 1.14; p < 0.01), basal plaque (OR 64.27; p < 0.01), low calcification (OR 0.06; P < 0.01) and high calcification (OR 0.03; P < 0.01) emerging as predictors of PC improvement (≥ 20°).

Notwithstanding this reported efficacy, long-term follow-up studies have been scantly published up to now^5^. A recent updated phase 4 study which included men who had received CCH during the IMPRESS I/II trials, reported that at 5-year assessment (*n* = 180), despite no additional treatment, there was a significant additional 9.1% improvement in terms of mean PC as compared with reference data^6^.

Of even more relevant clinical importance, there is no evidence to support that CCH increases the risk of these subsequent issues like shortening or erectile dysfunction (ED)^7^. As for penile PD-associated shortening, several strategies to optimize penile length in patients with PD have been reported. For instance, a recent systematic review showed that in men with a severe curvature but a preserved erectile function (EF), PD plaque incision (PIG) was advisable in order to maximize penile length, is spite of a greater risk of postoperative ED, recurrent curvature, or penile shortening^8^. Conversely, the impact of surgical procedure for penile curvature persistence after CCH is an important topic for both surgeons and patients. Thereof, aim of the current study was to investigate the surgical outcomes of patients with persistent PC after CCH.

## Materials and methods

### Study setting and patients

Complete medical records from 90 patients complaining of persistent PC after CCH treatment in a multicentre setting from 6 andrological centers between November 2018 and December 2019 have been retrospectively analysed.

The inclusion criteria were as follows: stable phase PD, with pain resolution and curvature stability for no less than three months; previous three injections of intraplaque CCH, according to the shortened protocols previously published by Abdel Raheem et al.^9^; a persistent penile curvature > 30° after CCH injections which negatively impacted toward sexual function.

All patients underwent a baseline, preoperative penile dynamic duplex ultrasound (DDU) to assess PC and EF. The interval between the last dose of CCH and surgical intervention was between 8 and 14 months. Likewise, all patients completed a preoperative International Index of Erectile Function-Erectile Function (IIEF-EF)^10^. According to PC and EF, patients were segregated into three groups: Group 1, including patients with PC > 60° and IIEF-EF < 17), who underwent PC correction by plaque incision grafting (PIG) with concomitant penile prosthesis implantation (PPI); Group 2, including patients with mild-moderate curvature (PC ≤ 60°) and IIEF-EF > 17, who have been submitted to PIG without PPI; and, Group 3 including patients with PC ≥ 60° but good EF (IIEF-EF > 17), who underwent Nesbit’s technique. All surgeries had been performed by senior andrologists.

The study was conducted according to the Declaration of Helsinki and protocol was approved by local ethical committee (University of Florence). All subjects provided informed consent.

### Surgical techniques

#### Graft with PPI

**Figure 1:**
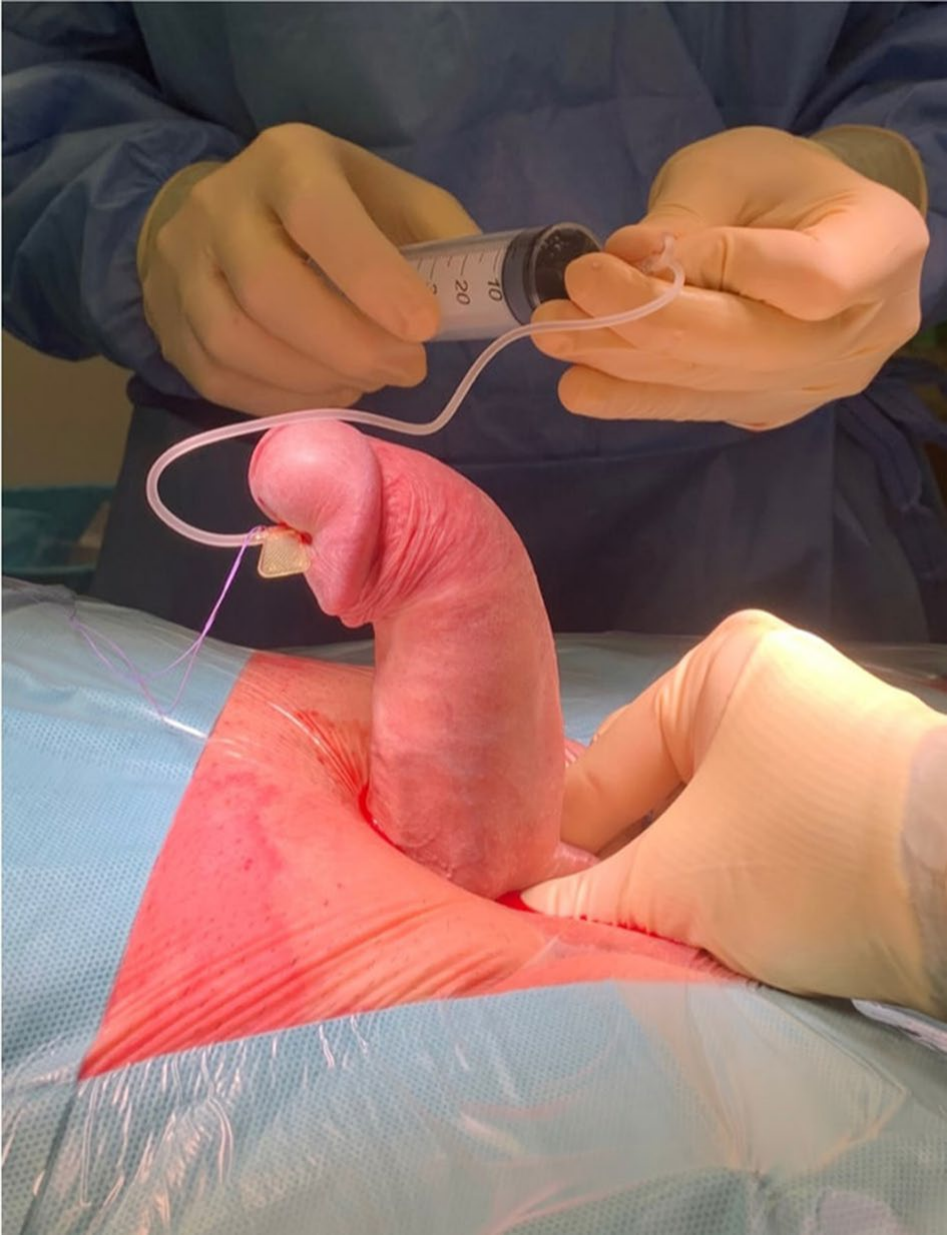
Identification of the maximum curvature point on the concave side of the penile shaft, through an artificial erection with saline solution.

**Figure 2:**
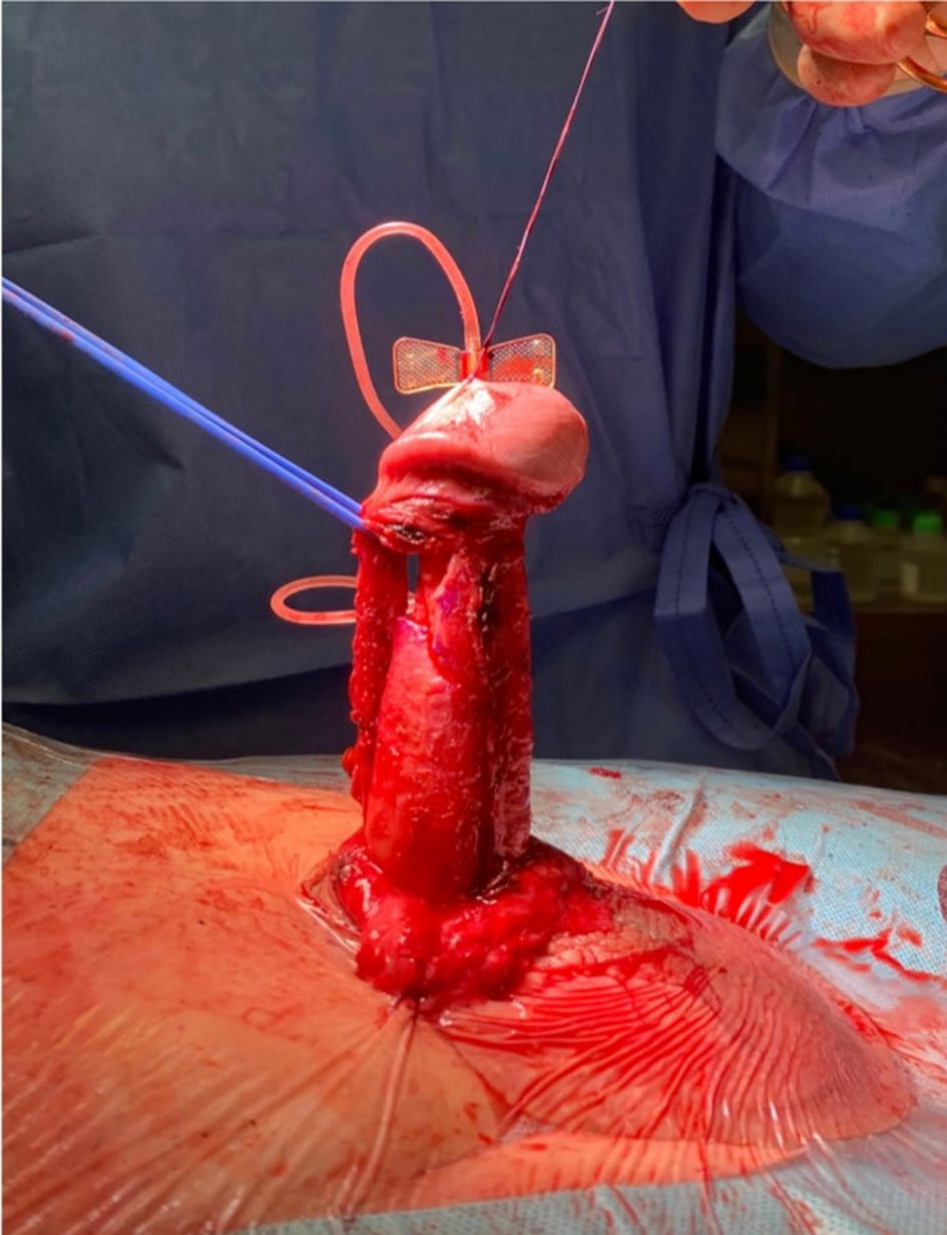
A standard double-Y relaxing tunical incision was performed to completely straighten the penile shaft.

**Figure 3:**
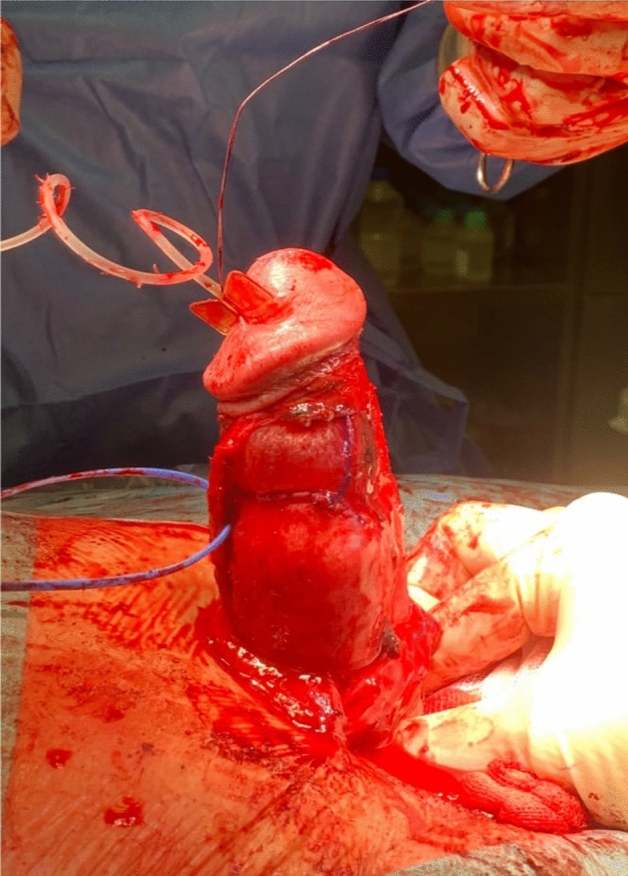
Application of penile grafting and fixation to the edges of the tunical defect with a 4-0 PDS running suture**.**

The penile shaft was degloved through a subcoronal incision. According to the direction of the curvature, the dorsal neuro-vascular bundle (NVB) was elevated through two para-urethral incisions in the case of a dorsal curvature, while the urethra was dissected from the corpora cavernosa in men with a ventral curvature. In order to precisely identify the point of maximum curvature on the concave side of the penile shaft, an artificial erection with saline solution was induced (Fig. 1). A standard double-Y relaxing tunical incision was performed to completely straighten the penile shaft (Fig. 2). Thereafter, the size of the albugineal defect was carefully measured and the graft shaped accordingly. In this context, either bovine pericardium graft [Collagen Matrix, Baxter Healthcare Corporation, CA, USA] or collagen fleece (Baxter Healthcare Corporation, CA, USA) have been used as grafting materials, based on surgeon's preference, according to previous report^11^. More in depth, when bovine pericardium graft was applied, the patch was oversized by 30% to prevent subsequent excessive contractures and fixed to the edges of the tunical defect with a 4-0 PDS running suture (Fig. 3). Conversely, when the collagen fleece was applied without suture, it was adequately shaped to obtain at least 1 cm overlapping on the tunica albuginea edges to promote an effective sealing. In both cases, a bilateral running closure of Buck’s fascia was performed for haemostatic purposes.

Moreover, an additional peno-scrotal incision was performed and the corpora cavernosa were isolated to allow a longitudinal corporotomy to be bilaterally carried out. Hence, dilation of the corpora cavernosa was obtained up to number 11 Hegar dilators, and the PPI was then completed. A dartoic scrotal pouch was created to insert the pump and the reservoir was placed into the Retzius space following Wilson’s technique (ectopic reservoir placement)^12^.

#### Graft without PPI

Similarly, a complete penile degloving was carried out through a subcoronal circumcising incision. Afterwards, as previously described, either the dorsal NVB or the urethra were dissected and an artificial erection was induced with saline solution to precisely identify and expose the point of maximum curvature. Likewise, an albugineal double-Y incision was performed on the concave side of the penile shaft. Following the aforementioned step, a graft was accurately applied on the site of substance loss. The surgical procedure was then concluded with a running suture to close Buck’s fascia and with the reconstruction of the penile layers. The same grating materials have been used, according to surgeon's preference.

### Nesbit

This procedure has been previously described^13^. The Nesbit technique involves excision of the tunica opposite the curvature without any plication.

### Postoperative management

An indwelling catheter, a suction drain and a penile compressive dressing were maintained at least over the first 24 h postoperatively. Hence, starting 2 weeks after surgery, a penile rehabilative protocol was applied, similarly with previous report^14^ but as follows: in Group 1, an early cycling of the implant was highly suggested; conversely, in Group 2 and 3, a penile stretching (i.e., pulling the glans manually or using external penile traction devices) along with and the regular intake of phosphodiesterase type 5 inhibitors (any type) were highly recommended to prevent/minimize penile shortening due to the scarring process.

### Main outcome measures

Data were retrospectively extracted from hospital medical records and postoperative outpatient visits. Hospital stay, operative time, postoperative complications and PC recurrence (defined as a persistent or recurrent PC > 20°) were selected as primary outcome measures. Overall satisfaction and further sexual functional outcomes were assessed using the validated questionnaires (IIEF-15, and the Peyronie's Disease Questionnaire (PDQ)^15^, which have been administered both preoperatively and at 3-mo postop assessment. Patients partners’ sexual satisfaction was also assessed before and after surgery with the Female Sexual Function Index (FSFI)^2,16,17^. Calcification level was classified as: absence of calcification; low perilesional calcification; and high calcification, modifying the previous definition by Levine et al.^4,18^. Penile length has been considered as the measurement from the pubis to the meatus.

### Statistical analyses

Continuous variables are presented as medians and interquartile range (IQR). Differences between groups were assessed using a Kruskall–Wallis or Mann–Whitney U test, as appropriate. Categorical variables were tested using a × 2 test or Fisher’s exact test. The chi-square test was used for categorical variables. The maintenance of penile curvature after surgery has been defined as “penile curvature persistence”.

Statistical analyses were carried out using STATA statistical software package (v.14), with a p-value of < 0.05 being considered significant.

## Results

Table 1 lists the baseline descriptive statistics for the whole cohort of patients. Of all, 25 (27.8%) patients received grafting procedure + PPI (Group 1), 18 (20.0%) patients were only submitted to grafting (Group 2), and 47 (52.2%) underwent the Nesbit technique only (Group 3). As for grafting materials, bovine pericardium graft and collagen fleece have been used in in 22 (51.2%) and 21 (48.8%) patients, respectively.

**Table 1:** Baseline descriptive statistics (n = 90).

Patients’ age (years)	56.5 (48.0–64.0)
Partners’ age (years)	54.0 (44.0–56.0)
Duration of the disease (months)	12.0 (10.0–16.0)
Penile curvature before CCH (°)	70.0 (60.0–90.0)
Penile curvature after CCH (°)	50.0 (45.0–60.0)
Penile length before surgery (cm)	15.0 (12.0–16.0)
IIEF-EF	21.0 (15.0–27.0)
PDQ-PS	9.0 (6.0–12.0)
PDQ-PP	6.0 (0.0–6.0)
PDQ-BD	10.0 (9.0–19.0)
FSFI	35.0 (30.0–38.0)
Diabetes [n (%)]	2 (2.22)
**Degree of calcification [n (%)]**	
None	36 (40.0)
Low	37 (41.11)
High	17 (18.89)
**Plaque site [n (%)]**	
Basal	45 (50.0)
Medial	19 (21.11)
Distal	26 (28.89)
**Direction of penile curvature [n (%)]**	
Dorsal	79 (87.78)
Ventral	7 (7.78)
Lateral	4 (4.44)

Data are expresses as medians (IQR).

*IQR *interquartile range, *IIEF *international index of erectile function, *CCH *collagenase *Clostridium histolyticum*, *PDQ *Peyronie’s disease questionnaire, *FSFI *female sexual function index.

At baseline, groups were similar in terms of age (p = 0.19), PDQ-PS (p = 0.89), PDQ-PP (p = 0.89), PDQ-BD (p = 0.29), and FSFI (p = 0.07), respectively. In contrast, groups differed for IIEF-EF (p < 0.01) and penile curvature (p < 0.01), as by definition of entry criteria. In particular, Group 3 patients had higher IIEF-EF (21.0; [interquartile range (IQR)] 18.0–24.0) respect to group 1 (18.0; IQR 16.0–20.0) and group 2 (17.0; IQR 16.0–20.0). Likewise, Group 3 (50°; IQR 45.0–50.0) and Group 2 (50°; IQR 45.0–50.0) patients had lower PC compared to Group 1 (62.5°; IQR 58.0–66.0). Groups did not differ in terms of duration of surgery (p = 0.62).

Table 2 shows the median improvement of clinical variables after surgery in comparison between groups. In particular, we reported a median penile length gain of + 2.0 cm (IQR 1.0, 2.0), + 1.0 cm (IQR 1.0, 1.0) in Group 1, 2 respectively and a loss of 2.0 cm (− 3.0, − 1.0) in Group 3.

**Table 2:** Median changes of clinical variables from baseline to final follow-up in the three groups.

	Group 1	Group 2	Group 3	p-value*
Penile length (cm), median (IQR)	+ 2.0 (1.0, 2.0)	+ 1.0 (1.0, 1.0)	− 2.0 (− 3.0, − 1.0)	< 0.01
IIEF-EF, median (IQR)	+ 2.0 (1.0, 2.0)	+ 2.5 (1.0, 4.0)	+ 2.5 (1.0, 4.5)	0.73
PDQ-PS, median (IQR)	− 3.0 (− 3.0, 2.0)	− 2.0 (− 3.0, − 1.0)	− 2.0 (− 2.5, − 1.0)	0.04
PDQ-PP, median (IQR)	− 2.0 (− 2.0, 0.0)	− 0.5 (− 2.0, 0.0)	− 0.5 (− 2.0, 0.0)	0.21
PDQ-BD, median (IQR)	− 3.0 (− 6.0, − 3.0)	− 3.0 (− 9.0, − 2.0)	− 3.5 (− 8.0, − 4.5)	0.35
FSFI, median (IQR)	4.0 (3.0, 5.0)	+ 4.5 (3.0, 5.0)	+ 4.0 (2.0, 5.0)	0.91

*Group 1 *grafting procedure + penile prosthesis implant (PPI), *Group 2 *grafting procedure only, *Group 3 *Nesbit procedure, *IQR *interquartile range, *IIEF *international index of erectile function, *PDQ *Peyronie’s disease questionnaire, *FSFI *female sexual function index.

*Kruskal–Wallis test.

Group 1 patients showed higher increase in penile length after surgery and a better improvement in terms of PDQ-PS score. In contrast, both IIEF-EF and FSFI scores did not differ among groups.

After surgery the median of overall satisfaction in all cohort was 8.0 (IQR 8.0–9.0) with no difference between groups (8.0 [IQR 8.0–9.0] in Group 1; 8.0 [IQR 3.0–9.0] in Group 2; 8.0 [IQR 8.0–9.0] in Group 3; p = 0.13).

In total, 86 (95.6%) patients did not report any surgical complication. Among all, 4 (4.4%) patients had PC persistence; of those, 2 (8.0%), 1 (5.6%) and 1 (2.1%) cases were observed in Group 1, Group 2 and Group 3, respectively. Patients of Group 1 and 2 report 100% of paresthesia.

## Discussion

Current findings suggest that grafting procedures with or without PPI or Nesbit technique were feasible, effective and safe to treat persistent PC after CCH injections, with PIG being superior in maintaining penile length compared to other surgical strategies.

Surgical success definition may vary between many studies, but the majority of authors defined it as any deviation as failure while some retained that a curvature less than 30° that does not prevent penetrative intercourse as a successful outcome^19^. However, studies reported different rates of success, from 54 to 100%^20^.

Obviously, it is important to set up a more standardized of success considering many aspects, including penile straightening and patient satisfaction. However, to maintain and to restore penile length are ideal, but the patient should be aware that erection after surgery is consistently different from those pre-operative and also before the onset of the PD-process^19^.

In line with our results, Bajic et al.^21^ recently reported a number of characteristics of patients who may eventually need to be operated after CCH for penile curvature persistence. In particular, patients with persistent bother after CCH treatment had high rates of indentation/narrowing, plaque calcifications, and mean composite curvature (MCC) > 60° at CCH treatment completion. Surgery is more common with hinge, but the intervention is safe and feasible in these patients, with low complication rates.

Similarly, DeLay et al. in a retrospective analysis of patients who had intralesional CCH treatment for PD and who subsequently underwent penile plication (PP), plaque incision and grafting (PIG), or inflatable penile prosthesis (IPP) placement reported efficacy of surgery at least 6 months after the last CCH injection^22^.

Levine et al. examined intraoperative and postoperative outcomes of a surgical correction in men with persistent PC after previous courses of intraplaque CCH. Of clinical relevance, no anatomical difficulties or complications secondary to a previous intraplaque CCH treatment has been reported during surgery. Intraoperative time was representative of a standard tunical plication and plaque excision graft surgeries (range 88–146 min). All men reported penile curvature < 20° post-surgery^23^. We have had the same experience, with no cases which had been either more difficult or complicated because of a previous intraplaque CCH injection.

It is also important to highlight that penile shortening is reported by most patients presenting with PD and represents one of the most bothersome symptoms^24^; a previous course of three intraplaque CCH injections did not appear to increase the risk of further penile shortening after surgery.

On the other hand, penile shortening after plication can be mitigated by the use of post-operative traction therapy and PDE5-I use^14,25^.

A further question was about the most correct strategy in terms of cost-efficacy of surgery (any technique) after CCH treatment. To this aim, Cordon et al. reported that the calculated probability of treatment success after injection was 49.5% for moderate curvature (30°–60°) and 12% for severe curvature (61°–90°) and that CCH appeared to be most appropriate for men with moderate, as opposed to severe, penile deformities^26^.

On the other hand, in the real-life setting patients often prefer more conservative therapies to surgery, at least at the beginning. In a 2017 survey of men with PD, only 18% of men ever chose surgery for management of their PD, at a mean 10.4 years after their initial diagnosis^27^.

As said, 5-year follow-up data from the IMPRESS I/II trials, suggested that patients undergoing CCH depicted significant improvements in terms of PC and satisfaction^6^. Vice versa, despite Sukumar et al. reported that injection therapies such as CCH are increasingly displacing surgical management as the primary treatment option for PD^28^, many patients may have insufficient improvement of their PC after intraplaque CCH injections, with a consequent negative impact on their quality of life.

Nevertheless, the surgical approach should not be considered as a retrograde step but a feasible and reliable option. The main problem of the surgical approach, as with the Nesbit’s procedure, remains penile shortening. This complication represents a significant problem for patients with a short preoperative penile length and may be caused by the progressive and diffuse fibrosis that may occur in PD. Indeed, penile curvature persistence after CCH may give way to plaque incision and grafting.

Surgery for penile curvature persistence after unsuccessful injections should take into account the prevention of penile length loss, the straightening of PC and the maintenance of post-surgery EF, which will probably become the gold standard approach for non-responders to minimally invasive treatment in the near future.

This study is not devoid of limitations. First, being a retrospective analysis, the study did not allow to include any randomization protocol among the three groups. Second, both the multicenter inclusion of patients and the heterogeneity of surgeons may represent a strength of the analyses, on the one hand, but on the other they may give rise to a major methodological flaw, even associated with the relatively small sample size. Third, the short follow-up (3-months) should be implemented with 12 months of follow-up in order to have stable results after this surgical procedure. Fourth, we included patients who received Raheem-modified CCH short protocol and for this reason our results should be reproduced in the setting of CCH standardized cycle. Finally, we did not quantify the grade of paresthesia after surgery and we also did not use published formula that evaluated degree of discrepancy causing penile curvature and to set strategy for dimensions of venous graft required for penile curvature correction^29^.

However, this study reports findings from the largest series ever published of surgery after intraplaque CCH injections persistence of penile curvature^30^.

## Conclusions

In the case of persistent bothersome penile curvature after intraplaque CCH treatment, surgical corrections by grafting with or without concomitant PPI or Nesbit technique represent a feasible, effective and safe option. No significant postoperative complications were recorded. Considering functional outcomes and patient satisfaction rate, surgery per se resulted in good overall satisfaction and sexual and general quality of life improvement.
